# The Buckeye Doctor—A Tale for Physicians and for Physicians’ Patients

**Published:** 1903-10

**Authors:** 


THE BUCKEYE DOCTOR—A TALE FOR PHYSICIANS AND FOR PHYSICIANS' PATIENTS.—By William W. Pennell, M. D. 345 pages. Price, $1.25. Published by The Grafton Press, New York.

This is a charming little story of a young physician who had "hung out his shingle" in an Ohio town, in competition with two old "quacks." A few cases in which the young man had been called in consultation by the villagers with the older doctors had shown to all concerned the immeasurable superiority of his modern methods. Their envy, hate and jealousy are shown in a thousand petty ways, but the climax of the story is reached in the arraignment and trial of the young doctor for "body snatching," in which he was the victim of foul play. He is, of course, cleared and restored to the confidence of the people. A sweet little love story runs through the whole and adds greatly to its interest.

It is a typical romantic novel, with its intensity of loves, friendships, hatreds, jealousies, its coincidents and rapidly changing scenes, and depends more upon its running narrative style for its pleasing qualities than upon any value as a character study. Two characters of opposite natures stand easily foremost—"Hank Crow," as an example of ignorant, malicious drunkenness, and

"Reuben Cary," that rough diamond whose whole-souled friendship will easily remain the pleasantest recollection of the story.

But it is much easier to criticise than to write, and we ourselves have enjoyed the story quite sufficiently to recommend it to all brother practitioners and friends who like a good story.

